# An initial game-theoretic assessment of enhanced tissue preparation and imaging protocols for improved deep learning inference of spatial transcriptomics from tissue morphology

**DOI:** 10.1093/bib/bbae476

**Published:** 2024-10-04

**Authors:** Michael Y Fatemi, Yunrui Lu, Alos B Diallo, Gokul Srinivasan, Zarif L Azher, Brock C Christensen, Lucas A Salas, Gregory J Tsongalis, Scott M Palisoul, Laurent Perreard, Fred W Kolling, Louis J Vaickus, Joshua J Levy

**Affiliations:** Department of Computer Science, University of Virginia, Charlottesville, VA 22903, USA; Emerging Diagnostic and Investigative Technologies, Department of Pathology and Laboratory Medicine, Dartmouth Health, Lebanon, NH 03766, USA; Emerging Diagnostic and Investigative Technologies, Department of Pathology and Laboratory Medicine, Dartmouth Health, Lebanon, NH 03766, USA; Department of Epidemiology, Dartmouth College Geisel School of Medicine, Hanover, NH 03756, USA; Program in Quantitative Biomedical Sciences, Dartmouth College Geisel School of Medicine, Hanover, NH 03756, USA; Emerging Diagnostic and Investigative Technologies, Department of Pathology and Laboratory Medicine, Dartmouth Health, Lebanon, NH 03766, USA; Thomas Jefferson High School for Science and Technology, Alexandria, VA 22312, USA; Department of Epidemiology, Dartmouth College Geisel School of Medicine, Hanover, NH 03756, USA; Department of Epidemiology, Dartmouth College Geisel School of Medicine, Hanover, NH 03756, USA; Emerging Diagnostic and Investigative Technologies, Department of Pathology and Laboratory Medicine, Dartmouth Health, Lebanon, NH 03766, USA; Emerging Diagnostic and Investigative Technologies, Department of Pathology and Laboratory Medicine, Dartmouth Health, Lebanon, NH 03766, USA; Genomics Shared Resource, Dartmouth Cancer Center, Lebanon, NH 03756, USA; Genomics Shared Resource, Dartmouth Cancer Center, Lebanon, NH 03756, USA; Emerging Diagnostic and Investigative Technologies, Department of Pathology and Laboratory Medicine, Dartmouth Health, Lebanon, NH 03766, USA; Emerging Diagnostic and Investigative Technologies, Department of Pathology and Laboratory Medicine, Dartmouth Health, Lebanon, NH 03766, USA; Department of Epidemiology, Dartmouth College Geisel School of Medicine, Hanover, NH 03756, USA; Program in Quantitative Biomedical Sciences, Dartmouth College Geisel School of Medicine, Hanover, NH 03756, USA; Department of Dermatology, Dartmouth Health, Lebanon, NH 03756, USA; Department of Pathology and Laboratory Medicine, Cedars Sinai Medical Center, Los Angeles, CA 90048, USA; Department of Computational Biomedicine, Cedars Sinai Medical Center, Los Angeles, CA 90048, USA

**Keywords:** spatial transcriptomics, deep learning, specimen preparation, whole slide imaging, data valuation

## Abstract

The application of deep learning to spatial transcriptomics (ST) can reveal relationships between gene expression and tissue architecture. Prior work has demonstrated that inferring gene expression from tissue histomorphology can discern these spatial molecular markers to enable population scale studies, reducing the fiscal barriers associated with large–scale spatial profiling. However, while most improvements in algorithmic performance have focused on improving model architectures, little is known about how the quality of tissue preparation and imaging can affect deep learning model training for spatial inference from morphology and its potential for widespread clinical adoption. Prior studies for ST inference from histology typically utilize manually stained frozen sections with imaging on non-clinical grade scanners. Training such models on ST cohorts is also costly. We hypothesize that adopting tissue processing and imaging practices that mirror standards for clinical implementation (permanent sections, automated tissue staining, and clinical grade scanning) can significantly improve model performance. An enhanced specimen processing and imaging protocol was developed for deep learning-based ST inference from morphology. This protocol featured the Visium CytAssist assay to permit automated hematoxylin and eosin staining (e.g. Leica Bond), 40×-resolution imaging, and joining of multiple patients’ tissue sections per capture area prior to ST profiling. Using a cohort of 13 pathologic T Stage-III stage colorectal cancer patients, we compared the performance of models trained on slide prepared using enhanced versus traditional (i.e. manual staining and low-resolution imaging) protocols. Leveraging Inceptionv3 neural networks, we predicted gene expression across serial, histologically-matched tissue sections using whole slide images (WSI) from both protocols. The data Shapley was used to quantify and compare marginal performance gains on a patient-by-patient basis attributed to using the enhanced protocol versus the actual costs of spatial profiling. Findings indicate that training and validating on WSI acquired through the enhanced protocol as opposed to the traditional method resulted in improved performance at lower fiscal cost. In the realm of ST, the enhancement of deep learning architectures frequently captures the spotlight; however, the significance of specimen processing and imaging is often understated. This research, informed through a game-theoretic lens, underscores the substantial impact that specimen preparation/imaging can have on spatial transcriptomic inference from morphology. It is essential to integrate such optimized processing protocols to facilitate the identification of prognostic markers at a larger scale.

## Introduction

### Emergence of spatial transcriptomics technologies and computational methods

For centuries, histological examination of tissue has been fundamental in disease prognostication [1]. Although such examination remains a cornerstone in pathology, the advent of genomic technologies has broadened our understanding of tumorigenesis, highlighting the value of examining expression patterns to gain comprehensive insights into tumor behavior and therapeutic response [2–4]. Typically, histopathological analysis is supplemented by immunohistochemical staining [5]. These evaluations provide spatial insights into molecular signatures that underscore cellular heterogeneity within a tissue sample. However, most immunohistochemical and fluorescence assays are limited in the number of markers they can analyze simultaneously. This limitation has been addressed with the emergence of spatial transcriptomics (ST) technologies, such as the Visium platform from 10× Genomics, which offers high multiplexing capability at remarkable spatial resolution, transforming our capacity to study expression patterns within intricate, nuanced tissue architectures [6, 7].

Recent methodological advancements in ST have concentrated on clustering spatial patterns of gene expression, detecting spatial variation, and mapping single-cell ribonucleic acid (RNA) sequencing data to specific tissue locations to identify cell sublineages and quantify cell types, thereby elucidating tissue architecture and cellular communication. Techniques integrating histology with ST have been developed to significantly enhance data resolution, effectively bridging morphological and molecular data to enrich our understanding of tissue biology and disease pathology from both molecular and morphological perspectives [6, 8–20].

### Impact of deep learning architectures and data quality on inference of spatial transcriptomics from histology

Our prior research has diverged from the aforementioned methods by inferring ST data from histological images through computational analysis. This strategy tackles the challenges of high costs and reproducibility issues associated with current ST assays by establishing a link between tissue morphology and molecular profiles, opening the door to scalable, low-cost multiplexing capabilities, where the morphology allows. Leveraging inferred expression patterns allows for downstream analyses comparable to traditional ST methods, circumventing the economic and technical limitations that typically constrain the widespread use of ST [6]. Current methods for inference of spatial transcriptomic patterns are inspired by virtual staining techniques [21–23], which use computational methods to predict molecular traits from routinely collected histological images. This obviates the need for further tissue staining/assaying. Preliminary studies have supported the potential of these techniques to expand highly–multiplexed spatial molecular evaluations to more extensive cohorts in a cost-effective manner. Initial studies on ST inference from histology have primarily emphasized enhancing deep learning architectures to boost performance. However, these efforts often overlook the importance of specimen processing and high-quality imaging, which are essential for clinical adoption. Effective predictive models hinge on the availability of high-resolution images with uniform staining, highlighting the critical need for standardized, automated staining protocols, and sophisticated imaging technologies. However, acquiring such quality images remains a challenge in many existing ST inference studies. As indicated in Supplementary Table 1, nearly all previous ST inference studies have utilized frozen tissue sections, stained manually, with imaging scanners which are not considered to be clinical grade or capable of scanning at 40× resolution [24–33]. For instance, frozen tissue sections are often used for rapid diagnosis or treatment of lesions intraoperatively. Frozen tissue sectioning often results in significant tissue artifacts (e.g. tears, bubbles, and folds) compared to formalin fixed paraffin embedded (FFPE; permanent) tissue slides and often the tissue morphology is challenging to distinguish as compared to permanent tissue sections with more pronounced tissue morphology. As frozen sections are not used in routine clinical diagnostic workflows, which may require spatial molecular analysis, algorithms developed using these slides may not generalized to permanent fixation and thus may have limited translational potential [34–37].

Aside from specimen fixation, leveraging whole slide image (WSI) scanners like Aperio GT450s to enhance whole slide image quality and ensuring uniform staining through automated processes is crucial for acquiring consistent and reliable input data [38]. This strategy, vital for precise alignment of imaging with omics data, offers the potential for more nuanced assessments of tissue histology, addressing the constraints imposed by manual staining and lower-resolution imaging techniques. By mitigating these limitations, it opens the possibility of identifying an expanded array of biomarkers directly from histological analysis. Moreover, increasing the sample size for training virtual ST inference algorithms is essential for encompassing a diverse array of patient and tumor characteristics without incurring additional costs, thus enabling more comprehensive analyses applicable to larger cohorts.

The recent introduction of the CytAssist device potentially addresses these concerns [39, 40]. In contrast to the traditional Visium FFPE assay, which mandates manual staining and specific Visium slide imaging conditions (loosely adhered coverslips and short imaging window), the CytAssist allows for the stable coverslipping of slides and extended time frame between staining, imaging, and analyte retrieval. In addition, the CytAssist protocol relies on tissue sections placed onto standard histology slides rather than costly Visium barcoded slides, simplifying tissue placement and allowing the selection of specific regions of interest for analysis. Together, this design allows multiple tissue sections to be more easily amalgamated onto a single slide before Visium profiling and facilitates the utilization of automated staining technologies and cutting-edge imaging using clinical-grade pathology infrastructure. These improvements not only augment whole slide image quality for intricate, deep learning analyses but might also considerably diminish associated costs.

### Study aim: data valuation to quantify the impact of enhanced specimen processing on performance of deep learning spatial transcriptomics inference models, compared to costs

In this research, we investigate the algorithmic performance benefits of utilizing the CytAssist device with enhanced protocols to improve data quality, aiming to discern if these improvements justify the associated costs. We employ a game theoretic approach, specifically the data Shapley method, to quantify the value and impact of tissue samples processed with these enhanced protocols on algorithmic performance compared to traditional methods. Quantifying the algorithmic benefits of data quality in this way allows us to directly assess the value of improved data quality against the costs of specimen processing to inform best practices. The data Shapley has been previously applied in machine learning and biomedical imaging to identify factors affecting predictive performance, often related to data quality. However, its application in this context offers a unique perspective on equitable data valuation for quantifying factors related to improved ST-inference beyond advances in deep learning architecture.

Our comparison centers around a cohort of colorectal cancer (CRC) patients. Colon cancer, increasingly affecting younger age groups, is a major global health issue due to its high prevalence and mortality. Crucial for determining prognosis and guiding treatment, colon cancer staging primarily relies on the Tumour, Node, and Metastasis (TNM) classification, which evaluates tumor invasion, lymph node involvement, and metastasis [41–44]. Notably, metastasis critically affects patient outcomes, marking increased tumor aggressiveness. While the TNM system is pivotal, it might not encompass the entire complexity of tumor biology, prompting research into additional molecular markers to enhance prognostic accuracy and predict metastasis/recurrence risks [45]. Studying the spatial distribution of specific genes within the tumor and its immune microenvironment can provide insights into antitumoral reactions, potentially enhancing colon cancer staging by identifying novel prognostic markers [46–48]. Our focus in this study is on assessing how specimen processing and imaging influence the accuracy of ST inference from histological images, rather than an exhaustive exploration of new markers. Future research will build upon our findings to conduct a comprehensive analysis with large-scale ST inference aimed at uncovering novel markers for tumor metastasis and recurrence.

**Table 1:** Description of patient cohort. Tissue sections from 13 patients (14 sections) are divided among 10 capture areas, with up to 2 tissue sections per capture area joined together (left/right side). *Indicates held-out capture areas 5 and 6 from serial sections from the same patient and location for testing. All other tissue sections come from different patients. MSS– Microsatellite Stability.

				Section placed on left side/center of capture area					Section placed on right side of capture area				
Protocol	Capture area	CytAssist	Dimension (mm^2)	Age	Sex	Tumor site	MSI status	METS	Age	Sex	Tumor site	MSI status	METS
Traditional: manual stain + low resolution imaging	1	no	6.5 × 6.5	40–45	F	Transverse colon	MSS	yes	–	–	–	–	–
	2	no	6.5 × 6.5	60–65	M	Right colon	MSS	no	–	–	–	–	–
	3	no	6.5 × 6.5	80–85	M	Right colon	MSS	yes	–	–	–	–	–
	4	no	6.5 × 6.5	45–47	F	Right colon	MSS	no	–	–	–	–	–
	5*	yes	6.5 × 6.5	80–83	M	Left colon	MSI	yes	–	–	–	–	–
Enhanced: automated staining + high resolution imaging	6*	yes	6.5 × 6.5	80–85	M	Left colon	MSI	yes	–	–	–	–	–
	7	yes	11 × 11	90–95	F	Hepatic flexure	MSI	yes	80–85	M	Left colon	MSI	no
	8	yes	11 × 11	85–90	F	Splenic flexure	MSS	yes	75–80	F	Hepatic flexure	MSS	no
	9	yes	11 × 11	80–85	F	Cecum	MSI	yes	70–75	M	Cecum	MSI	no
	10	yes	11 × 11	55–60	F	Left colon	MSS	yes	65–70	M	Sigmoid	MSS	no

## Methods

### Data collection

*Specimen overview:* Our dataset comprises specimens processed through two distinct protocols: the traditional protocol (four patients, four capture areas) and the new enhanced protocol (eight patients, four capture areas). In addition, two paired serial sections were profiled for comparative analysis, the first emulating the traditional protocol and the second mirroring the enhanced protocol.

*Patient and capture area selection:* This dataset represented thirteen patients diagnosed with pathologic T Stage-III (pT3) CRC. These patients were selected through a retrospective review of pathology reports from 2016 to 2019. Four patients were featured in a previous study where we restricted these patient characteristics based on microsatellite stable tumors and tumor site (right/transverse colon) [24]. For the remaining cohort of nine patients, to ensure a balanced representation of patient characteristics, the patients were matched based on various criteria, including age, sex, tumor grade, tissue size, and mismatch repair/microsatellite instability (MMR/MSI) status [49]. MSI status was determined by assessing the loss of expression of mutL homolog 1 (MLH1) and postmeiotic segregation increased 2 (PMS2) proteins through immunohistochemistry. Tissue blocks were sectioned into 5–10-micron thick layers, and specific regions of interest such as epithelium, tumor-invasive front, intratumoral areas, and lymphatics. Capture areas were annotated by a pathologist from WSI taken at serial tissue sections. Representative regions were carefully dissected from the tissue, placed into capture areas, and subjected to hematoxylin and eosin (H&E) staining, imaging, and Visium profiling in the Pathology Shared Resource at the Dartmouth Cancer Center and Single Cell Genomics Core in the Center for Quantitative Biology.

**Figure 1:**
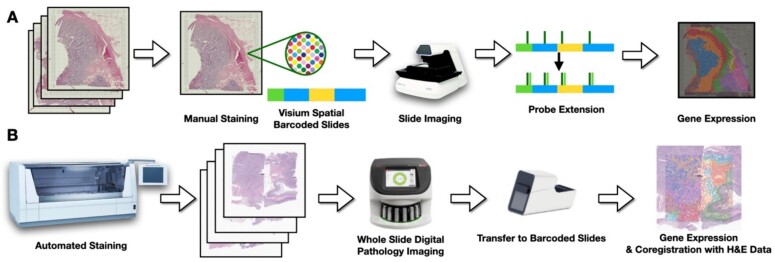
Comparative overview of the two protocols. (a) Traditional protocol: after placing tissue on Visium barcoded slide, sections are manually stained with H&E and imaged using the EVOS m7000. (B) Enhanced protocol: automated application of chemical reagents with 40× resolution imaging via Aperio GT450, followed by transfer to Visium device facilitated by 10× CytAssist.

**Figure 2:**
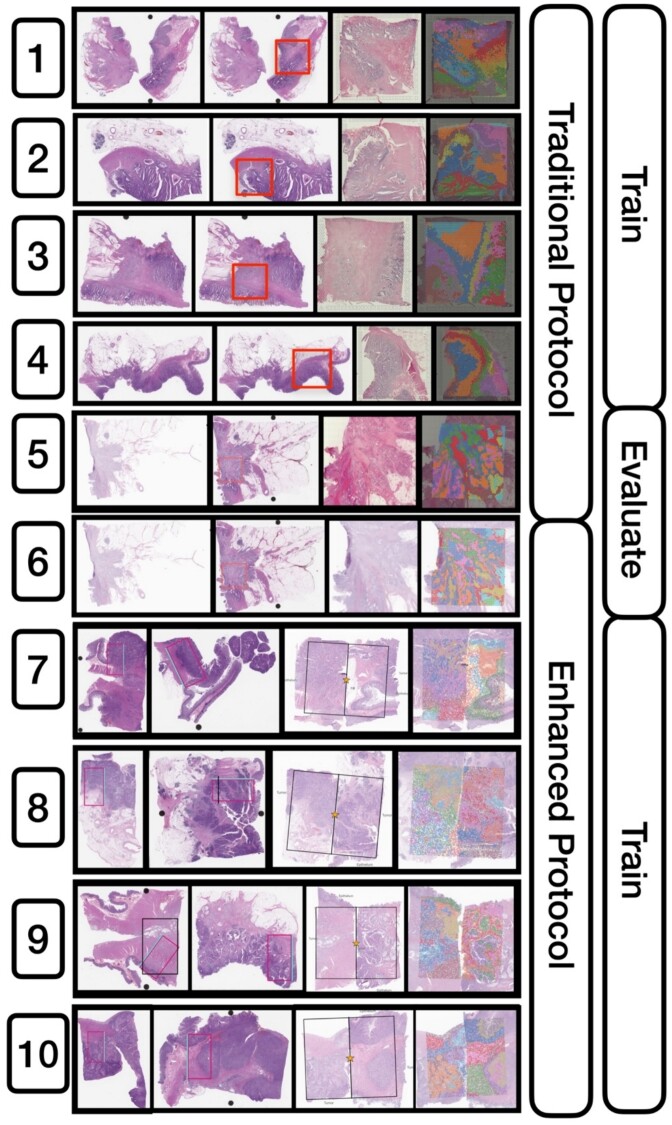
Detailed configuration of the 10 capture areas and their specimen processing and imaging protocol. The figure presents the progression of all 10 capture areas, from their inception to visualization. The sequence is as follows: (i) H&E-stained slide, (ii) selected capture area dedicated to one or two patients, (iii) visualization of capture area, and (iv) integration with ST clusters. Training involved the use of either traditional, enhanced, or a combination of both slide types. Notably, capture areas 5 and 6 were set aside as held-out serial sections for subsequent analyses.

*Traditional protocol:* The first four capture areas (capture areas 1–4; Table 1, Figs 1a and 2) were profiled using the traditional 10× Visium FFPE protocol—after macrodissection, placement onto the Visium barcoded slide, and manual H&E staining, the Visium protocol: (i) images the tissue at 10–20× resolution using standard image scanning (EVOS m7000 scanner, Thermo Fisher); (ii) the tissue is permeabilized for hybridization of whole transcriptome messenger RNA (mRNA) probes; followed by, (iii) probe ligation and release for capture on the Visium slide through poly(A) tail binding; next, (iv) captured probes are extended and amplified to incorporate spatial barcodes; and (v) the probes and spatial barcodes are sequenced on an Illumina NovaSeq instrument [50] targeting 50 000 reads/spot. The 10× Genomics SpaceRanger software is used to convert raw sequencing data into spatially-resolved gene expression matrices. This comprehensive process enables whole transcriptome (mRNA) profiling of up to 5000 55µm spots with a 100µm center-to-center distance within a 6.5 mm^2^ capture area or 14 000 spots within an 11 mm^2^ capture area. After post-filtering uninformative reads, we obtained ~17 943 genes at ~5000 locations for each slide (total Visium spots: 4950, 4922, 4887, and 4169 per slide).

*Enhanced protocol:* We trialed an improved specimen processing protocol designed specifically for profiling specimens with the Visium CytAssist assay. This protocol integrates the CytAssist technology with improved specimen processing within a pathology department to ensure consistent staining and optimal image quality by leveraging the capabilities of automated slide stainer via the Sakura Tissue-Tek Prisma Stainer (Sakura Finetek USA, Inc. 1750 West 214th Street, Torrance, CA 90501) [51] and the Aperio GT450s imaging system. The imaging was conducted at a high resolution of 40× (equivalent to 0.25 μm per pixel) before proceeding with Visium profiling. Four tissue slides were collected representing eight patients, resulting from macrodissection of tissue sections from FFPE blocks. Patient selection criteria were well-matched to the set used for the traditional protocol save for MSI status and age, which featured additional variation in this expanded set of patients. These sections were precisely marked by pathologists in serial WSIs to target specific tissue architectures. To maximize resource efficiency, tissue segments from two patients were merged onto a single slide, creating an 11 × 11 mm capture region. This strategy ensured each capture area contained an equal representation of metastasis and MSI status from anatomically similar sites. Using our improved protocol, we first (i) placed FFPE tissue sections onto standard histology slides, followed by coverslipping in a glycerol + xylene mounting medium, (ii) performed deparaffinization, rehydration, and H&E staining on a Leica Bond instrument, (iii) collected WSI at 40× resolution on Aperio GT450 scanners, and (iv) decoverslipped in xylene for 1–3 days (until coverslips were detached). The remaining steps of destaining, probe hybridization, probe ligation, eosin staining, transfer to the Visium slide using CytAssist, and library preparation were performed according to the manufacturer’s protocol (CG000485). Libraries were sequenced on an Illumina NovaSeq targeting 50 000 reads/spot. This detailed method permits unbiased gridded profiling of spots within slides area. The subsequent imaging of the same tissue slide (after staining with eosin) facilitated precise co-registration of the 40× high-resolution pathology slide with the Visium ST. After the manual selection of fiducials, the Spaceranger software was employed to align CytAssist sections with their corresponding 40× H&E stains, which ensures accurate co-registration, and conduct quality control and convert the Visium ST data into an easily interpretable format (Table 1, Figs 1B and 2). We obtained expression profiles at around 7000 locations for each patient (total Visium spots: 7696, 6640, 6956, 7380, 6881, 6421, 7261, 6159, and 4778 per patient). It should be noted that this enhanced protocol does not yet apply to fresh frozen sections (see Supplementary Table 1).

**Figure 3:**
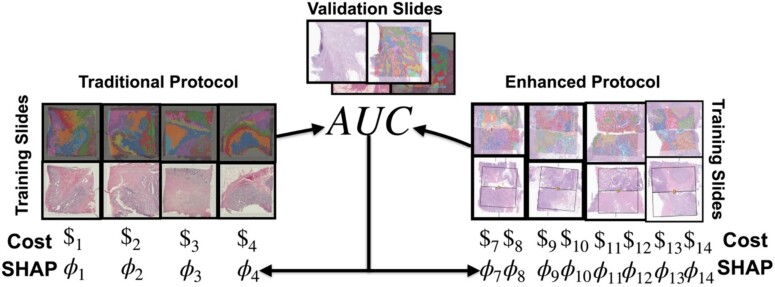
Experimental design for data valuation/cost comparison: data Shapley values were calculated for individual patients in the cohort to represent marginal algorithmic performance gains. The data Shapley value for each slide was compared to the costs associated with specimen assaying.

*Comparison slides:* To mitigate the influence of inherent tissue variability and rigorously assess the CytAssist technology, we restricted validation of the deep learning models two serial sections from the same patient as comparison points to evaluate our machine learning models. Serial sections were spatially matched/scored to represent identical capture areas with nearly identical histological and molecular features. Capture Areas 5 and 6 were earmarked for our comparative analysis of the CytAssist technology (Table 1, Figs 2 and 3). Each slide from these areas underwent distinct preparation methods to mirror both the traditional and our enhanced protocol. From these slides, the set of nearly 18,000 genes was reduced to the 1000 most spatially variable genes using the SpatialDE package for direct comparison of model performance based on training data acquired with these protocols [52].

### Machine learning modeling and evaluation

For WSI acquired using the enhanced protocol, co-registered 40× WSI corresponding to each Visium slide were cropped into 512 × 512 pixel sub-images centered on each Visium spot within the capture areas, selected based on a previous study that had conducted a sensitivity analysis over various patch sizes [24]. For WSI acquired using the traditional protocol, each Visium spot encompasses a circular capture zone with a 130-pixel diameter at a 20× magnification. Several deep learning models—utilizing an Inceptionv3 convolutional neural network architecture as described in previous work—were trained to predict Visium ST at each spot for both binary (i.e. low/high expression, dichotomized by median expression) and continuous (e.g. log-transformed of pseudo counts with an offset of 1 read) prediction tasks for 1000 spatially-variable genes. Models were trained using the mean squared error on the log-transformed counts for continuous data. As aforementioned, performance for dichotomous tasks was calculated through dichotomization via median expression [53, 54]. Model parameters were selected based on optimal performance statistics across an internal validation set across the training epochs. Hyperparameters were set based on a coarse hyperparameter search for each method.

### Experimental comparisons

We used a comprehensive comparative analysis to discern the benefits of collecting data using the enhanced protocol against the traditional protocol. The experimental comparisons probed the protocols under various training and validation regimes, thereby providing insights into their relative strengths and potential synergies (Table 2). We evaluated our models using paired serial sections from both protocols, ensuring minimal tissue variability to highlight the impact of staining and imaging methods. The performance comparisons were based on the training sets described in Table 2.

### Performance evaluation

*Evaluative assessments on comparison slides:* Spot level expression was compared on the held-out comparison slides, retaining their native imaging resolutions. Direct evaluation focused on recapitulating the expression at each individual Visium spot across the entirety of the held-out slides. This granular assessment ensured an in-depth understanding of how well the trained models can predict expression profiles at localized regions throughout the slides. Confidence in model performance is reported through 95% confidence intervals derived from 1000-sample non-parametric bootstrapping of Visium spot observations.

*Performance metrics:* Performance metrics include the following: (i) quantitative metrics, area under the receiver operating characteristic curve (AUC) for dichotomous tasks and Spearman correlation for continuous tasks, macro-averaged across all genes. (ii) Qualitative evaluation, beyond quantitative scores, we examined the capability of each approach to mirror true expression patterns. This involved comparing the clustering of true expression patterns on those predicted from the tissue histology, utilizing the AlignedUMAP dimensionality reduction technique to generate visually comparable low-dimensional embeddings [55, 56]. A more effective method should ideally maintain the uniqueness and structure of the original clusters. To quantitatively assess our approach, we developed a k-nearest neighbors (k-NN) classifier trained on the embeddings of ground truth Visium ST spots to determine their cluster memberships. This classifier was then applied to the aligned embeddings of the inferred expression data for the same Visium ST spots, with the goal of replicating the original cluster memberships based on their proximity to the k-nearest ground truth expression spots. We conducted this analysis using both k = 3 and k = 5.

*Differential expression:* As another comparison between the traditional and enhanced protocol, we aimed to determine how well models trained on data from each protocol could localize biological markers to each tissue architecture, as compared to localization via the true expression patterns. Specifically, each Visium spot was annotated according to its location—either within the tumor, at its periphery, or distal to the tumor. We hypothesized that the enhanced slides would yield gene expression profiles more reflective of these distinct tissue regions, approximating the precision of the ground truth expression more closely than the traditionally processed slides would. To test this, we employed the Mann–Whitney U-test to analyze differential gene expression (treating expression as a continuous count–based measure) between the tumor-interface zones and those regions either within or away from the tumor [57]. This analysis focused on the top 200 genes as ranked by the Spearman correlation statistics between the true and predicted expression. We then compared the U-statistics obtained from the actual expression data to those generated from predicted expression, summarizing the results as the median percentage change in U-statistics across the examined genes, with 95% confidence intervals reported using 1000-sample non-parametric bootstrapping.

*Superresolution:* Several prior works for ST inference from histology have demonstrated the capabilities to infer ST at sub-spot resolution without the aid of any ST data. We have accomplished superresolution on held out tissue slides through inference on overlapping 512-pixel (~128 micron) tissue patches. Inferences were made in 128-pixel increments (~32 micron) [6, 8–20].

### Quantifying the impact of enhanced protocols on algorithmic performance versus assay cost through equitable data valuation

In this ST deep learning study, we aimed to quantify the algorithmic performance gains attributed to each slide. The data Shapley represents the individual contribution of inclusion of specific data point on the overall predictive accuracy of our models, as reflected by the AUC. Prior data valuation research has demonstrated that the quality of input data significantly affects assigned data Shapley values, reinforcing the relevance of our approach. The data Shapley was calculated by adopting a Monte Carlo method, which assigned a distinct data Shapley value to each slide in our dataset [58–60].

**Table 2:** Comparative analysis of model training approaches across distinct protocols. This table delineates the methodologies used for generating training data from both the traditional and enhanced protocols. Emphasis is placed on evaluating the implications of different staining and imaging methods. By employing reserved comparison samples, models trained on each dataset undergo assessment using paired serial sections from both protocols, aiming to reduce tissue-related variability

Training data	Purpose	Predictive analysis
Traditional protocol slides	To establish a foundational performance baseline	This comparison gaged the ability of models trained on the traditional method to predict spot-level expression across both techniques using the paired serial sections (encompassing both traditional and improved protocols).
Enhanced protocol slides	To spotlight the enhanced capability and superiority of the improved protocol	Evaluating predictions on the paired serial sections from both protocols showcases how models, when trained sections assayed through the improved protocol, interpret results from both techniques. Ideally, its performance should meet or surpass the traditional protocol’s metrics on the shared paired slides.
Slides from both protocols	To harness the collective merits of both protocols, forging a holistic understanding of tissue histology across various specimen processing and imaging methods	Training on tissue acquired from both protocols promises to impart a broader representation of the data to the models. Evaluations on the comparison slides reflect the model’s adaptability, informed by both protocols.

The formula used for the data Shapley value, based on the work of Ghorbani et al. (2019), is:


$$ {\phi}_i=\frac{1}{n!}\sum_{\pi \in \Pi}\left[V\left({S}_{\pi}^i\cup \left\{i\right\}\right)-V\left({S}_{\pi}^i\right)\right] $$

Here, $V(S)$denotes the AUC score for a set of slides $S$, ${S}_{\pi}^i$ is the set of slides preceding slide *i* in permutation $\pi$, and $\Pi$ represents the uniform distribution over all permutations. This equation evaluates the marginal test AUC increase when a training slide is included, thus gauging its importance. All training slides, regardless of protocol, were used for in the Monte Carlo method. This equitable data valuation enabled a direct comparison between the algorithmic benefits and the fiscal costs associated with different slide processing/imaging protocols. In calculating the costs, we rigorously reviewed all financial transactions tied to specimen profiling, including reagent, sequencing, and labor costs, disregarding any potential discounts to present a realistic expense scenario. Similar to the data Shapley estimates, these fiscal costs were calculated on a slide-by-slide basis, varied with the number of detectable spots on each slide, also depending on whether they were processed using traditional or enhanced protocols (Fig. 3). Mann–Whitney U-testing was used to compare enhanced and traditional protocols for training slides for: (i) specimen assaying costs, (ii) data Shapley when evaluating on a validation slide prepared with the enhanced protocol, (iii) data Shapley when evaluating on a validation slide prepared with the traditional protocol, and (iv) data Shapley-to-cost ratios for the aforementioned comparisons. Rank biserial correlations and Mann–Whitney *P*-values were used to communicate the effect sizes and statistical significance of associations [61].

## Results

### Enhanced staining and imaging protocol results in substantial boost in predictive performance

**Figure 4:**
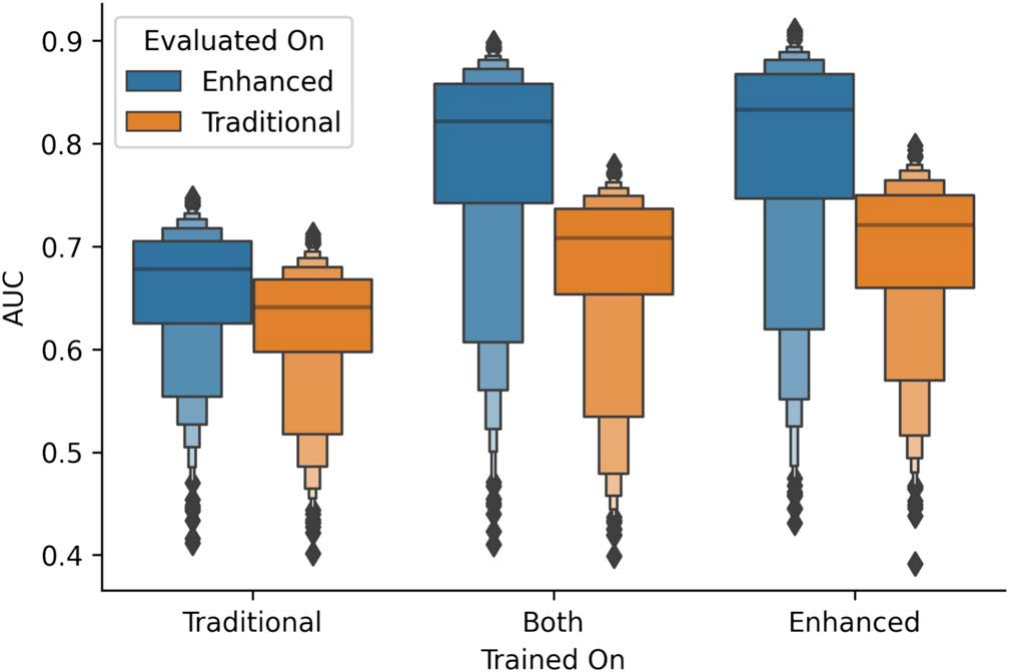
Boxenplot of AUC performance across top 1000 genes. This plot showcases the comparative performance of held-out capture areas based on training slide type (traditional, enhanced, or both) and evaluation slide type (traditional or enhanced), using the area under the receiver operating characteristic curve as the performance metric.

**Figure 5:**
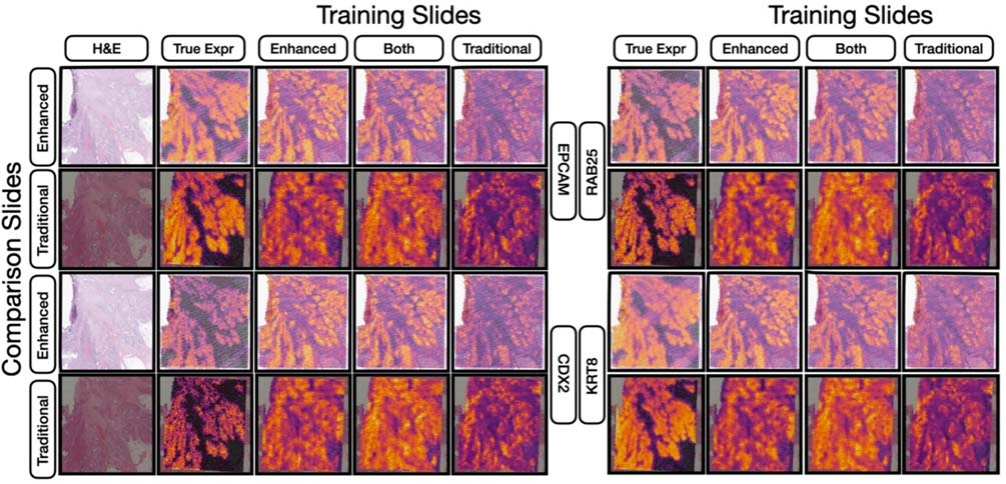
Heatmap visualization of gene expression predictions for four randomly selected genes. This figure juxtaposes the ground truth gene expression heatmaps against the predictions made by neural networks trained on either enhanced slides, both preparation approaches or traditional slides. Each prediction is showcased for both comparison slide types (traditional and enhanced). Specific markers from the held-out slides (both enhanced and traditional) are highlighted to emphasize the nuanced differences across training techniques and their evaluation.

When assessing the predicted expression against the true expression for the top 1000 spatially variable genes in the held-out slides, the models demonstrated remarkable accuracy, reported using AUC, root-mean squared error and Spearman statistics (Table 3, Fig. 4, Supplementary Fig. 1, Supplementary Table 2). Overall, models trained using on data acquired with the traditional protocol predicted expression on both traditional and enhanced slides with ~0.66 AUC and a 0.28 correlation. Use of the traditional protocol for acquiring training data was related to diminished performance. In contrast, exclusively leveraging the enhanced protocol led to a major increase in predictive performance. Specifically, while training and testing on traditional slides yielded an AUC of 0.641 and a 0.243 correlation, the same process on enhanced 40×-resolution WSI (enhanced protocol) catapulted the results to an AUC of 0.833 and a 0.625 correlation—this translates to a surge of nearly 45% in AUC and a staggering 157% in Spearman correlation. Through a pathway analysis (enrichR, Reactome database), the top 25 genes that could be predicted with high accuracy for models trained/evaluated with each protocol corresponded to a range of biological pathways previously implicated in colon cancer (Supplementary Data 1) [62]. Our exploration into whether a hybrid training approach, incorporating both traditional and enhanced slides, would augment performance turned out to be inconclusive, as it did not notably elevate predictive power for either slide type and instead lead to modest reductions in performance from training solely on WSI acquired through the improved protocol. As depicted in Fig. 5, visually, the expression patterns across a slide appear more clearly distinct and align more closely with the ground truth when training is conducted using enhanced slides, irrespective of whether the comparison slide utilized enhanced or traditional techniques. To address the potential impact of image resolution on our algorithmic findings, we downsampled all WSI to 10× resolution and separately compared model performance based on training data from the two protocols. Findings continued to support performance improvements relating to automated tissue staining (Supplementary Table 3).

**Table 3:** Performance metrics for held-out capture areas across top 1000 genes. This table presents the median AUC and spearman correlation coefficients and their respective 95% confidence intervals derived from a 1000-sample non-parametric bootstrap. Metrics are shown for each combination of training slide type (traditional, enhanced, or both) and evaluation slide type (traditional or enhanced)

Trained on	Evaluated on	AUC ± SE	Spearman ± SE
Traditional	Enhanced	0.678 ± 0.002	0.323 ± 0.003
Traditional	Traditional	0.641 ± 0.002	0.243 ± 0.003
Both	Enhanced	0.822 ± 0.003	0.605 ± 0.005
Both	Traditional	0.708 ± 0.002	0.389 ± 0.004
Enhanced	Enhanced	0.833 ± 0.003	0.625 ± 0.006
Enhanced	Traditional	0.72 ± 0.002	0.406 ± 0.004

### Comparing clustering fidelity, tissue architecture specificity for inferred spatial transcriptomics expression, and demonstration of superresolution

Similar to previous works [24, 53], we sought to understand whether slides processed using the enhanced protocol yielded predicted expression patterns that were clustered similarly as the ground truth expression, demonstrating capacity to replicate underlying biological variation and spatial expression heterogeneity (Supplementary Fig. 2, Supplementary Table 4). Using the k-NN classifier, we assessed the clustering fidelity of predicted expression patterns by examining if the inferred Visium spots preserved the same relative positioning in the embedding plot as the ground truth data, comparing models trained on both enhanced and traditional slides. The analysis showed that on two held–out serial section WSIs, models trained with the enhanced protocol more accurately matched cluster assignments for the corresponding enhanced–protocol validation slide (recovery proportion of 0.911 ± 0.115). Conversely, models trained on traditional protocols showed a decline in clustering accuracy on the enhanced-protocol test slide (recovery proportion of 0.878 ± 0.151). In contrast, for the validation slide processed using the traditional protocol, cluster assignments were most effectively replicated by models trained with traditional protocols (recovery proportion of 0.913 ± 0.113). Additional findings can be found in Supplementary Table 4.

We also evaluated each method’s capability to predict expression patterns characteristic of the tumor invasive margin in contrast to regions inside and distant from the tumor. Given the potential variability in predicted expression scales, we employed the Mann–Whitney U test to contrast expression across these tissue structures, documenting the percentage shift in U-statistics between actual and predicted expression. These findings are consistent with our predictive performance observations, suggesting that the ability to accurately predict expression is synonymous with more refined delineations of tissue architecture. This predictive performance and precision in the subsequent differential expression analyses are notably enhanced by the CytAssist leveraging the enhanced protocol (Supplementary Table 5). For example, models either trained on both enhanced and traditional slides or exclusively on enhanced slides were most successful in recapitulating the U-statistics derived from actual expression data when assessed on enhanced slides. However, there was a marked drop in accuracy when these metrics were applied to the reserved traditional slide.

Algorithms trained on slides using any paired histological and ST data are also capable of inferring spatial gene expression patterns at a greater resolution than the original data, without the aid of Visium ST data. We refer the reader to several examples where we applied the Inceptionv3 model trained on data acquired using the enhanced protocol on two held–out, intact tissue slides (Supplementary Figs 3 and 4). Tissue staining was automated and WSI were captured at 40× resolution, mirroring the enhanced protocol. The quality of these WSIs are comparable to a real-world clinical setting. Superresolution was achieved by inferring expression patterns on overlapping patches, taken at 128-pixel increments, rather than 220-pixels (55-micron at 40×) or 512 pixels (original stride length for large image analysis). The resolution can be increased through further reduction in stride length during inference, among other resolution-enhancing methods.

### Evaluating the trade-offs between specimen processing enhancements and costs via algorithmic performance metrics through data valuation

**Figure 6:**
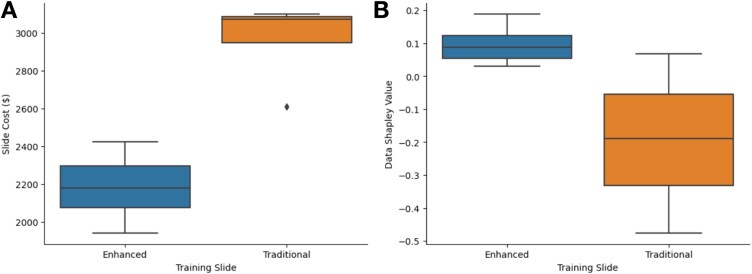
Boxplots illustrating data valuation and slide cost depending on whether training slide was prepared using the enhanced or traditional protocol: (a) slide costs were higher for slides prepared using the traditional protocol, and (b) data Shapley values were higher for slides prepared using the enhanced protocol.

In assessing cost-effectiveness, we considered the data Shapley values in conjunction with the assay costs, noting that some slides produced negative values, indicative of a reduced model performance. Upon analysis of the performance on the validation slide processed with the enhanced protocol, we found that training slides processed with the enhanced protocol generally demonstrated positive data Shapley values, suggesting beneficial contributions to the model (Fig. 6; Supplementary Figs 5 and 6; Supplementary Tables 6 and 7), whereas traditional protocol training slides more frequently incurred negative values (*P* = .028). Our analysis also revealed that when evaluating our models on a validation slide processed with the enhanced protocol, training slides processed using the enhanced protocol exhibit a higher average data Shapley-to-cost ratio compared to those processed with the traditional protocol (*P* = .008). The opposite held-true for data valuation based on the validation slide processed with the traditional protocol—training slides processed with the same protocol were valued more highly for enhancing predictive performance (*P* = .004, .004; Fig. 6; Supplementary Figs 5 and 6; Supplementary Tables 6 and 7).

## Discussion

Our study aimed to evaluate the influence of specimen processing in the realm of ST inference from histology. Here, we compared data collected using traditional and enhanced protocols for the task akin to virtual staining—a technique that can infer spatial expression patterns directly from whole slide image histomorphology [21, 63]. This method offers the potential to democratize ST insights to more extensive cohorts for genes exhibiting high predictability, subsequently broadening the spectrum of markers under consideration. Prior studies featuring spatial inference algorithms mostly leveraged frozen tissue sections that were manually stained and imaged with lower resolution scanners, presenting potential challenges in applying algorithms trained on this data for clinical–grade digital pathology workflows, which typically rely on permanent sections, consistent staining and high-resolution scanning. The central thrust of this study was to better understand and quantify the impact of data quality on algorithmic performance for clinical–grade virtual inference workflows. Enhancements in specimen preparation, specifically in three key areas including: (i) tissue multiplexing to reduce costs based on their positioning in the mounting medium, (ii) optimizing staining procedures, and (iii) refining imaging processes could facilitate more accurate image-based RNA inference and other integrative analysis, thereby boosting statistical precision. The incorporation of CytAssist was pivotal, offering insights into how upstream enhancements in specimen processing can yield vastly improved in silico outcomes. Data valuation helped quantify and compare the algorithmic benefits and fiscal costs of adopting the enhanced protocol versus the traditional protocol.

*Principal findings in the context of improved tissue staining:* This study underscores the discernible performance variations between slides processed through enhanced and traditional protocols, reflecting differences in tissue staining and imaging in the context of deep learning applications for ST [64]. Tissue staining, a technique to enhance the contrast between various tissue components, is of paramount importance. Dyes such as hematoxylin and eosin, with their distinct optical absorption properties, offer a range of color variations. When oxidized, hematoxylin interacts with various metals, forming complexes that produce unique colors, enhancing the dye’s staining capabilities [64–72]. Even minor deviations in staining procedures and timing can result in fluctuating staining intensity. Human variations in the timing of staining and the use of reagents nearing their expiration or when overused/over-oxidized/deteriorated can further compromise quality. Factors such as contaminants also introduce inconsistencies, affecting the uniformity of tissue staining. Automated staining solutions offer a promising alternative to manual methods, eliminating human-induced sources of variation. By standardizing the application of H&E stains according to a set protocol, both the quality and consistency of specimens can be enhanced. Removing these variations allows algorithms to shift their focus from capturing variability to representing the underlying structures with greater fidelity [66]. Past research validates that digital image analysis is frequently compromised by inconsistent staining. However, automating this process has been shown to not only improves staining consistency but also bolsters the contrast in tissue structures, thereby increasing diagnostic reliability [64–72]. For algorithms to effectively discern gene-related histologies, consistently capturing intricate details, better represented by minimizing these sources of variability, is vital.

Thus, it is unsurprising that tissues stained through automated processes exhibited superior performance. The models trained on tissue sections processed using the enhanced protocol demonstrated remarkably stronger predictive accuracy, as evidenced by higher AUC, Spearman correlation values and data Shapley values. Evaluation of these models on enhanced slides also presented a performance advantage, even when only training on traditionally processed slides. Moreover, we showcased that heightened predictive accuracy can lead to a bolstered statistical power in evaluating tissue architecture with enhanced slides relative to their traditional counterparts. Analysis of the aligned UMAP embeddings demonstrated that enhanced slides tended to yield expression patterns that clustered similarly to the ground truth and quantitative data valuation demonstrated that the algorithmic benefits conferred by collecting high quality input data far outweighed the costs.

*Interpretation of findings and the need for broader validation:* Our research affirms that by prioritizing specimen preparation and imaging, especially with the aid of CytAssist, one can amplify the statistical acuity of subsequent analyses and more authentically capture the intricate relationships among Visium spots from histological observations [30, 73]. This heightened precision, made possible by the enhanced staining and imaging protocol, has the potential to illuminate the molecular intricacies and spatial configurations of unique tissue structures. Such insights pave the way for a more profound comprehension of CRC metastasis, especially when these state-of-the-art techniques are applied to broader cohorts. By quantifying the importance of tissue processing and imaging through data valuation, we can potentially identify tissue of sufficient quality for developing clinical grade workflows for ST inference from histology at scale.

*Challenges and future directions:* This study focused on comparing enhanced and traditional protocols within a specific set of capture areas. The derived insights offer a foundational framework for both validating and scaling these techniques to expansive cohorts. However, there are a few considerations that warrant further attention. Firstly, the comparison between traditional and enhanced protocols was limited to a specific set of capture areas, necessitating further exploration to broaden the application, scope, and impact of our findings. However, the set of capture areas selected is not outside of what has been done for prior studies in this domain and the number of Visium spots used to train and validate the models far exceeds prior ST inference works. While we sought to reduce potential batch effects for various representative histologies featured in the study through standardizing staining/imaging, we acknowledge the potential for batch effects in ST data that can impact model training. Generalizing our findings to other tissue types, molecular pathways, and experimental setups should be further explored. For instance, we assessed performance on the top 1000 spatially variable genes—while this is a commonly adopted method for ST inference works, the selection of these genes may have influenced predictive performance. However, selection of varied gene sets and pathways for comparisons was outside of the scope of this work and will be explored in a future work. In addition, the enhanced protocol does not yet apply to fresh frozen sections, which will be the subject of future work. We did not compare the performance of various deep learning architectures as this task has been done in many prior works (see Supplementary Table 1) and is outside of the scope of the current study though is a consideration for future works expanding data valuation comparisons in the context of ST inference. To affirm the universality and adaptability of the models, varied staining methodologies, slide preparations, and tissue specimens should be considered, requiring additional forms of validation (e.g. immunostaining, alternative spatial transcriptomic assays) [74–76]. Such disparities can introduce unpredicted variability, with potential ramifications on model efficiency. Although there are algorithmic solutions for standardizing staining agents, a holistic approach may require a collaborative multicenter framework, strategies to alleviate batch inconsistencies, and close coordination among key stakeholders within various shared resource infrastructures across each institution [77]. Enhancing the scope of validation for deep learning paradigms, as well as identifying areas for improvement outside of algorithmic development (e.g. specimen processing), can catalyze the more widespread integration of these nascent ST technologies. The relevance of these findings hinges on external validation through independent cohorts. Moreover, the implications of our study should be expanded to encompass other diseases that warrant spatial molecular assessments [78–80]. Future applications of data valuation can identify tissue features likely to improve/reduce performance of ST inference approaches. For instance, tissue can become distorted after whole slide imaging prior to ST profiling. This can introduce tissue artifact that could significantly degrade algorithmic performance if unaccounted for—slides with these artifacts or unwanted histological features (e.g. mucinous tumors) can be identified with a data valuation approach. Future work will apply such methods at scale to further improve the quality of spatial inferences and will also examine application of valuation methods in the context of other computational approaches for ST.

## Conclusion

The validation of ST information inferred from WSI provides a unique opportunity to assess spatial molecular factors pertaining to CRC metastasis, recurrence and survival with greater statistical precision. Deep learning spatial inference methods frequently rely on large volumes of specimens to yield significant results, which can prove costly for spatial transcriptomic assays. Yet, the spatial molecular data inferred through our enhanced protocols, enhanced through sophisticated specimen processing, potentially diminish the necessity for such expansive and expensive datasets. Data valuation techniques can enhance spatial inference workflows by precisely quantifying the instances where tissue quality and imaging features align with or diverge from the standards required for clinical-grade analysis. Accurate extrapolations of gene expression landscapes within tissue samples can enable a more refined exploration of CRC metastasis, further emphasizing our imperative to further validate these approaches in larger cohorts.

**Key Points**

- This study showcases an enhanced protocol for preparing/imaging tissue for deep learning-based ST inference from histology, using the flexibility of the Visium CytAssist assay to facilitate improvements in tissue processing and imaging (enhanced protocol). Previous work typically performed ST inference on frozen tissue sections, manually stained, and imaged using non-clinical grade pathology image scanners.
- Leveraging Inceptionv3 neural networks on slides from 13 pT3 stage CRC patients, the enhanced protocol significantly outperformed traditional protocols in preparing tissue for accurate prediction of gene expression patterns. Inferring on slides processed using the enhanced protocol yielded gene expression profiles, which clustered similar to the actual data and identified relevant tissue architectures and biological pathways.
- By applying these models on external slides lacking ST data, ST profiles could be identified at higher resolution than the original Visium assay. The use of models trained on high-quality data on large, external cohorts prepared with clinical-grade digital pathology practices has the potential to enhance the statistical precision in pinpointing biomarkers associated with metastasis and recurrence.
- Data valuation techniques demonstrated that the algorithmic benefits of enhancing tissue/image quality far outweighed the fiscal costs. Data valuation has the potential to identify high quality, relevant WSI for ST inference tasks.
- Effective specimen processing is paramount for high-fidelity results in ST-driven cancer research. Working together, histotechnicians, pathologists, and genomics specialists play a critical role in advancing tissue preparation and imaging techniques, deepening our insight into tumor biology for further prognostic biomarker development.

## Supplementary Material

Enhanced_Workflow_supplement_accept_revisions_final_bbae476

suppl_data_1_bbae476

## Data Availability

Access to manuscript data is limited due to patient privacy concerns. All data produced in the present study are available upon reasonable request. Requests should be directed to senior author Dr. Joshua Levy (email: joshua.levy@cshs.org).
