# Supplementary material for: An initial game-theoretic assessment of enhanced tissue preparation and imaging protocols for improved deep learning inference of spatial transcriptomics from tissue morphology
Source: Brief Bioinform. 2024 Oct 4;25(6):bbae476. doi: 10.1093/bib/bbae476 (PMC11452536; doi:10.1093/bib/bbae476)
Supplement: Enhanced_Workflow_supplement_accept_revisions_final_bbae476 [file enhanced_workflow_supplement_accept_revisions_final_bbae476.docx]

## Supplementary Materials

**Supplementary Table 1: Review of Specimen Fixation, Staining and Scanning for Spatial Transcriptomics Inference Methods from Histology**

| Name/  Author | Method | Dataset | Patients | Genes | Spots | Fixation | Staining | Clinical-Grade Image Scanning | Citation |
| --- | --- | --- | --- | --- | --- | --- | --- | --- | --- |
| Fatemi-2023 | Inceptionv3, ViT, GNN | CRC pT3 | 4 | 1000 | 18928 | FFPE | Manual | No | [1] |
| Hist2ST | Transformer/GNN | HER2/  cSCC | 32/  12 | 785/  134 | 9612/  6630 | Frozen | Manual | No | [2] |
| HisToGene | ViT | HER2 | 32 | 785 | 9612 | Frozen | Manual | No | [3] |
| ST-Net | CNN | Breast | 23 | 102 | 30612 | Frozen | Manual | No | [4] |
| BLEEP | ResNet50 | Liver | 4 | 3467 | 9269 | Frozen | Manual | No | [5] |
| DeepHis2Exp | GNN | Breast | 45 | 1012 | Unclear | Frozen | Manual | No | [6] |
| Srinivasan-2023 | ViT | Skin | 4 | 1000 | 11124 | FFPE | Automated | Yes, 40X | [7] |
| DeepSpaCE | VGG16 | Breast | 3 | 24 | 14141 | Frozen | Manual | No | [8] |
| BrST-Net | ResNet50/Inceptionv3/  EfficientNet/ViT | Breast | 23 | 237 | 30612 | Frozen | Manual | No | [9] |
| THItoGene | CapsNet/ViT/GNN | HER2/  cSCC | 32/  12 | 785/  134 | 9612/  6630 | Frozen | Manual | No | [10] |
| CellGNN | GNN | CRC pT3 | 13 | 1000 | 83961 | FFPE | Automated | Yes, 40X | [11] |
| Present Work | Inceptionv3 | CRC pT3 | 13 | 1000 | 83961 | FFPE | Automated | Yes, 40X |  |


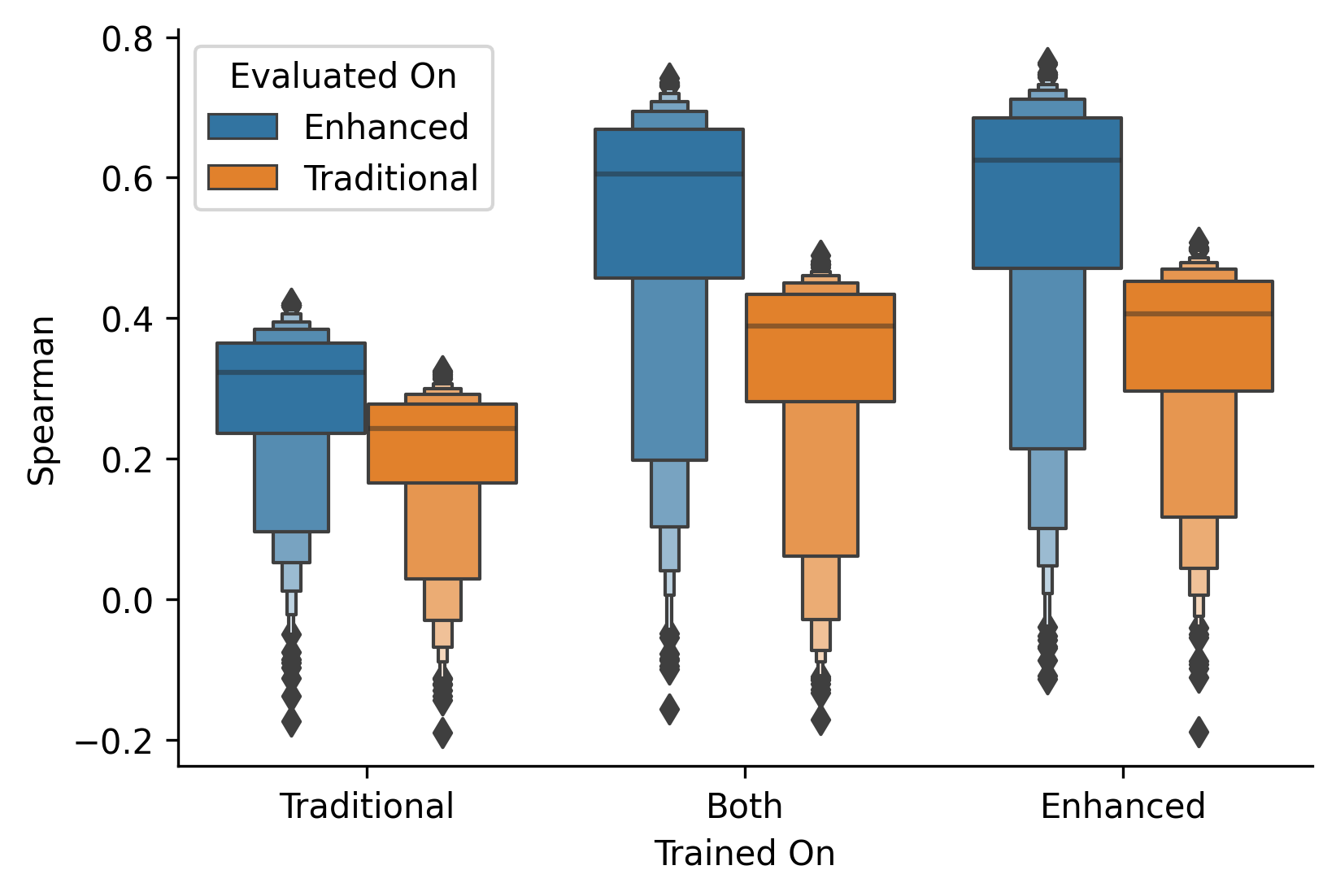


**Supplementary Figure 1: Boxenplot of Spearman Performance Across Top 1000 Genes.** This plot showcases the comparative performance of held-out capture areas based on training slide type (traditional, enhanced, or both) and evaluation slide type (traditional or enhanced), using the Spearman correlation coefficient as the performance metric.

**Supplementary Table 2: Root Mean Squared Error (RMSE) Performance Statistics of Predicted Pseudo-Log Expression for Training and Validating on Whole Slide Images Acquired through Various Protocols:** 1000-sample bootstrapped standard errors were calculated to compare the performance when traditional or enhanced protocols are used in training and validation of slides. Results were reported at different ranges of transcript abundance to account for potential heteroskedasticity in findings.

|  |  | Evaluation Data | |
| --- | --- | --- | --- |
| Expression Counts | **Training Data** | **Enhanced** | **Traditional** |
| 0-20 | Enhanced | 0.725±0.006 | 0.758±0.007 |
|  | Traditional and Enhanced | 0.772±0.005 | 0.809±0.006 |
|  | Traditional | 1.203±0.012 | 0.999±0.009 |
| 20-45 | Enhanced | 1.459±0.028 | 1.466±0.022 |
|  | Traditional and Enhanced | 1.645±0.024 | 1.568±0.023 |
|  | Traditional | 2.692±0.015 | 2.409±0.027 |
| 45-60 | Enhanced | 1.79±0.035 | 1.758±0.049 |
|  | Traditional and Enhanced | 1.935±0.022 | 1.757±0.051 |
|  | Traditional | 3.236±0.026 | 2.92±0.045 |
| 60+ | Enhanced | 2.029±0.03 | 1.986±0.049 |
|  | Traditional and Enhanced | 2.222±0.034 | 1.973±0.076 |
|  | Traditional | 3.614±0.018 | 3.284±0.047 |

**Supplementary Table 3: Performance Statistics for Training and Validation on Low Resolution Whole Slide Images -** This table presents the Area Under the Receiver Operating Characteristic Curve (AUROC) for training and spatial transcriptomics inference on whole slide images (WSIs) fixed at 10X resolution (1 micron per pixel; accomplished through downsampling). 1000-sample bootstrapped standard errors were calculated to compare the performance when traditional or enhanced protocols are used in training and validation of slides.

|  | | Validation Slide | |
| --- | --- | --- | --- |
|  |  | **Enhanced** | **Traditional** |
| Training Slides | **Enhanced** | 0.7568±0.0052 | 0.7279±0.0054 |
|  | **Both** | 0.7473±0.0043 | 0.7109±0.0053 |
|  | **Traditional** | 0.6746±0.0037 | 0.6894±0.0045 |

**Qualitative Assessment of UMAP Clustering for Various Specimen Preparation Protocols**

For evaluations conducted on the enhanced WSI, models trained using both enhanced and traditional slides together, as well as those trained solely on enhanced slides, produced the most pronounced clustering (**Figure 5**). The relative placements of these clusters closely reflected the ground truth. Conversely, the resulting clusters were less defined when models were assessed on the traditional slide. Notably, models trained on traditional slides seemed to conflate multiple clusters.


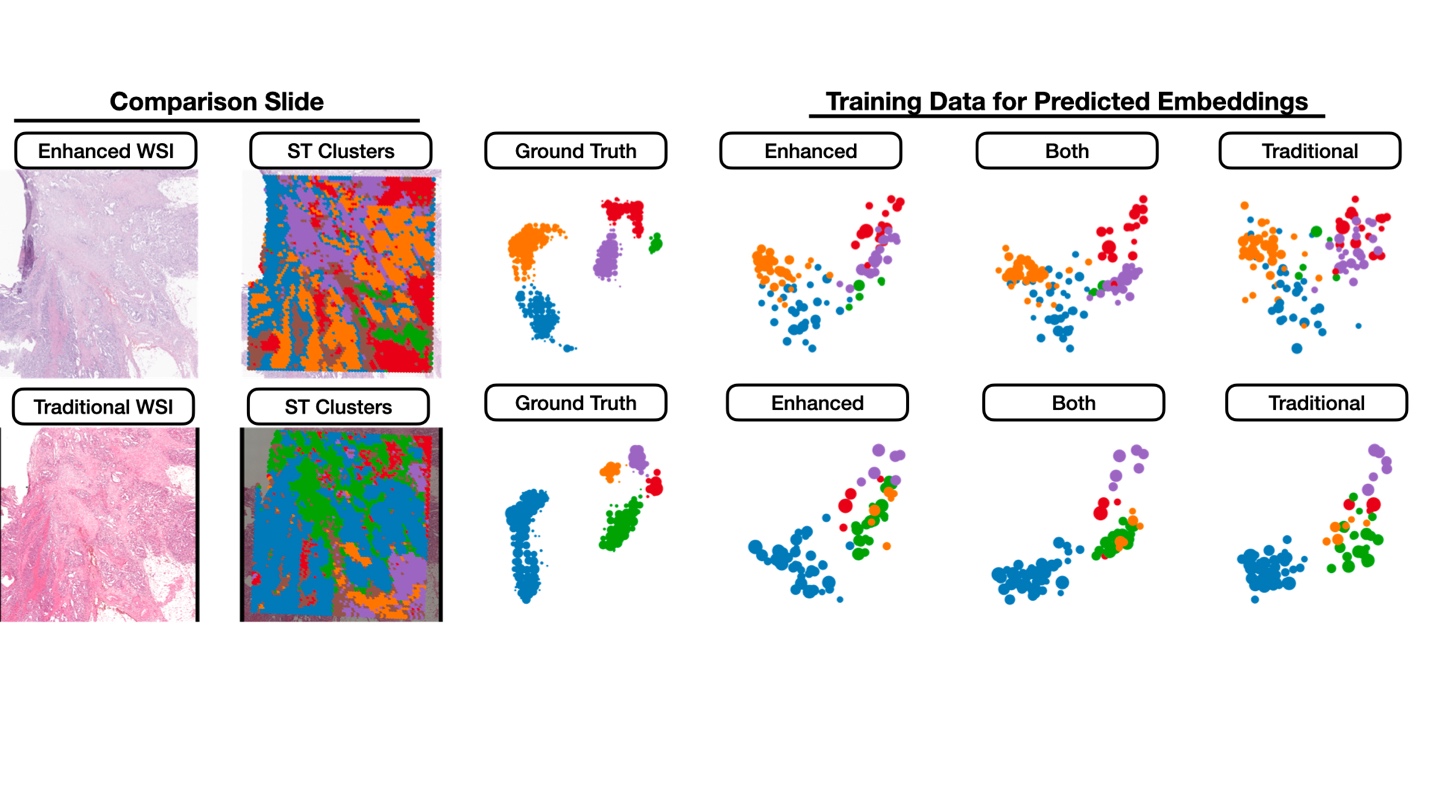


**Supplementary Figure 2: UMAP embeddings of true and predicted gene expression for enhanced and traditional evaluation slides.** The Mapper algorithm was used to organize the embeddings into groups with overlapping Visium spots, facilitating the visualization of continuous transitions between transcriptional states. Node sizes in the Mapper plots reflect the number of Visium spots, while colors represent the dominant cluster membership. These clusters were determined using AlignedUMAP embeddings followed by HDBSCAN clustering on the ground truth count-based expression data. Mapper was chosen for this analysis because it effectively reveals key, hidden relationships within the data while being robust to noise. It is particularly suitable for reducing the number of points to highlight the relative proximity between clusters. The algorithm defines nodes by overlapping Visium spots to capture continuous transitions, smooth out variations and noise, and prevent spurious cluster assignments. This approach enhances the discovery of meaningful relationships within the complex dataset. The use of overlapping points in Mapper is essential to accurately represent continuous transitions and relationships in the data. It ensures that nodes capture relevant structures without abrupt boundaries, reflecting the natural variability and density of the data. This overlapping also helps mitigate the loss of information that can occur with non-overlapping methods, providing a more nuanced and comprehensive visualization of the dataset. Mapper and other topological methods have previously been employed to summarize key relationships in single cell and spatial datasets [12–16].

**Supplementary Table 4: Cluster Replication Accuracy Using k-Nearest Neighbors Classification–** This table details the performance of a k-nearest neighbors (k-NN) classifier in replicating the cluster memberships of Visium ST spots from Aligned UMAP embeddings. The classifier, trained on ground truth data embeddings, was tested on the aligned embeddings of inferred expression data with the aim of preserving the unique structure of original clusters. Classification accuracy is reported for k values of 3 and 5, reported based on what protocol was used to acquire slide image(s) for training/validation.

| Evaluation Slide | Training Slide | K=3 | K=5 |
| --- | --- | --- | --- |
| Enhanced | Enhanced | 0.832 ± 0.198 | 0.911 ± 0.115 |
|  | Both | 0.827 ± 0.202 | 0.905 ± 0.121 |
|  | Traditional | 0.775 ± 0.247 | 0.878 ± 0.151 |
| Traditional | Enhanced | 0.737 ± 0.274 | 0.800 ± 0.227 |
|  | Both | 0.704 ± 0.295 | 0.764 ± 0.255 |
|  | Traditional | 0.864 ± 0. 167 | 0.913 ± 0.113 |

**Supplementary Table 5: Comparative Performance on Tumor Interface Markers Localization:** This table showcases the percentage variations in U-statistics between true and predicted gene expression (continuous count data) for the top-200 genes, emphasizing the method's precision in identifying expression differences at the tumor invasive margin relative to both its internal and surrounding areas. 95% confidence intervals were derived from a 1000-sample non-parametric bootstrap to measure the robustness of these findings. A diminished percentage difference signifies a heightened capability to pinpoint molecular markers at the tumor interface in a manner similar to the actual expression.

| Trained On | Evaluated On | U-Statistic Percent Change (%) | 2.5% CI | 97.5% CI |
| --- | --- | --- | --- | --- |
| Traditional | **Enhanced** | 18.73 | 18.42 | 18.93 |
| Traditional | **Traditional** | 14.19 | 13.79 | 14.43 |
| Both | **Enhanced** | 5.23 | 4.80 | 5.61 |
| Both | **Traditional** | 17.56 | 17.33 | 17.84 |
| Enhanced | **Enhanced** | 5.40 | 5.22 | 5.77 |
| Enhanced | **Traditional** | 17.11 | 16.90 | 17.47 |

**
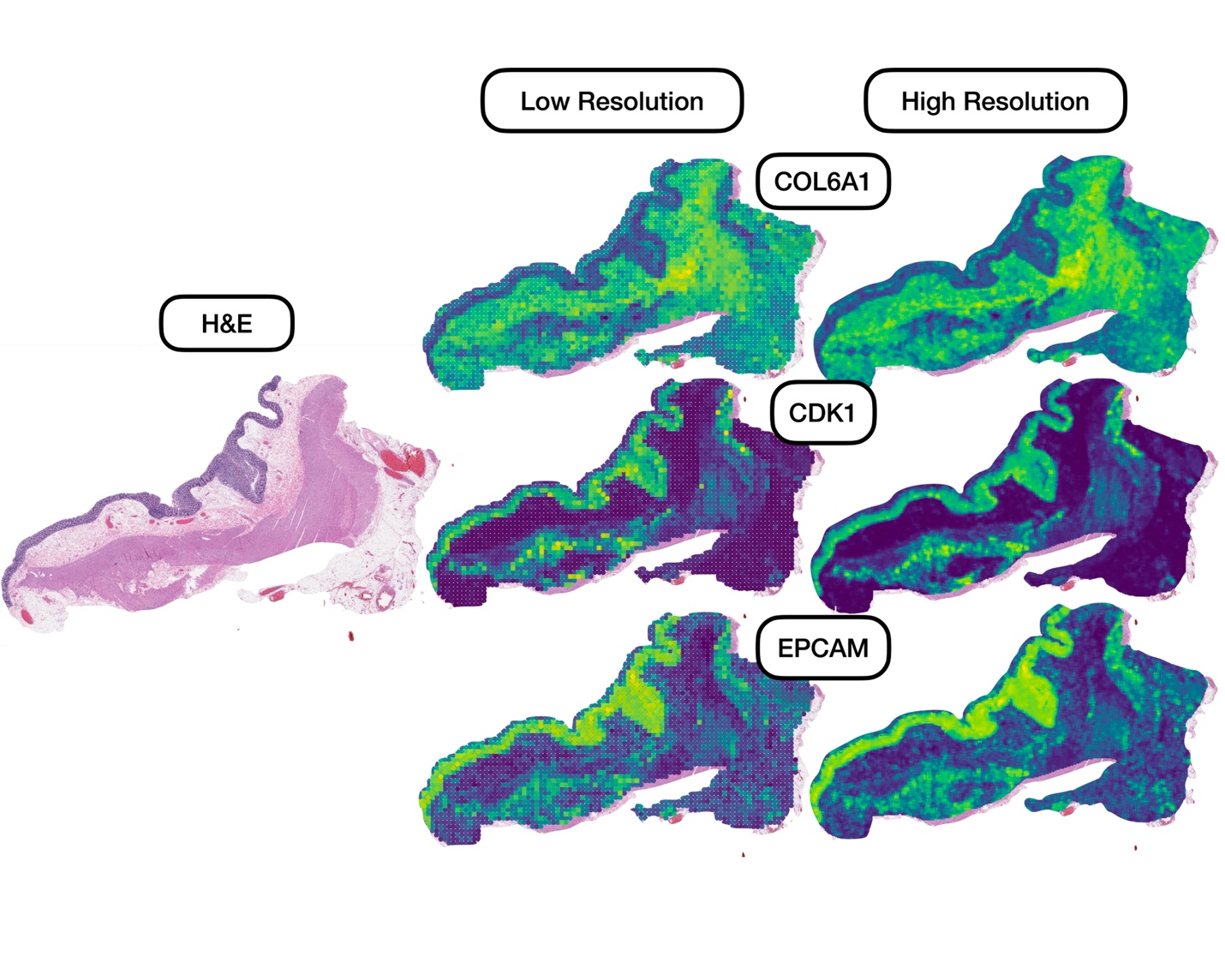
**

**Supplementary Figure 3: High-Resolution Inference of Expression Patterns for T0 CRC patient (held-out slide)–** This figure demonstrates the capacity to extrapolate expression patterns at resolutions surpassing those of the original model training. Utilizing the versatility of imaging features, which can be extracted from any location on the slide, we have conducted inferences at varying resolutions. For low resolution, analysis was performed with non-overlapping 512-pixel strides and patches, whereas high resolution involved overlapping 128-pixel strides, with the same subimage dimensionality. The figure specifically visualizes the expression of COL6A1, indicative of collagen activation, CDK1, a cyclin-dependent kinase associated with tumor activity, and EPCAM, an epithelial cell marker.

**Top of Form**

**Bottom of Form**

**
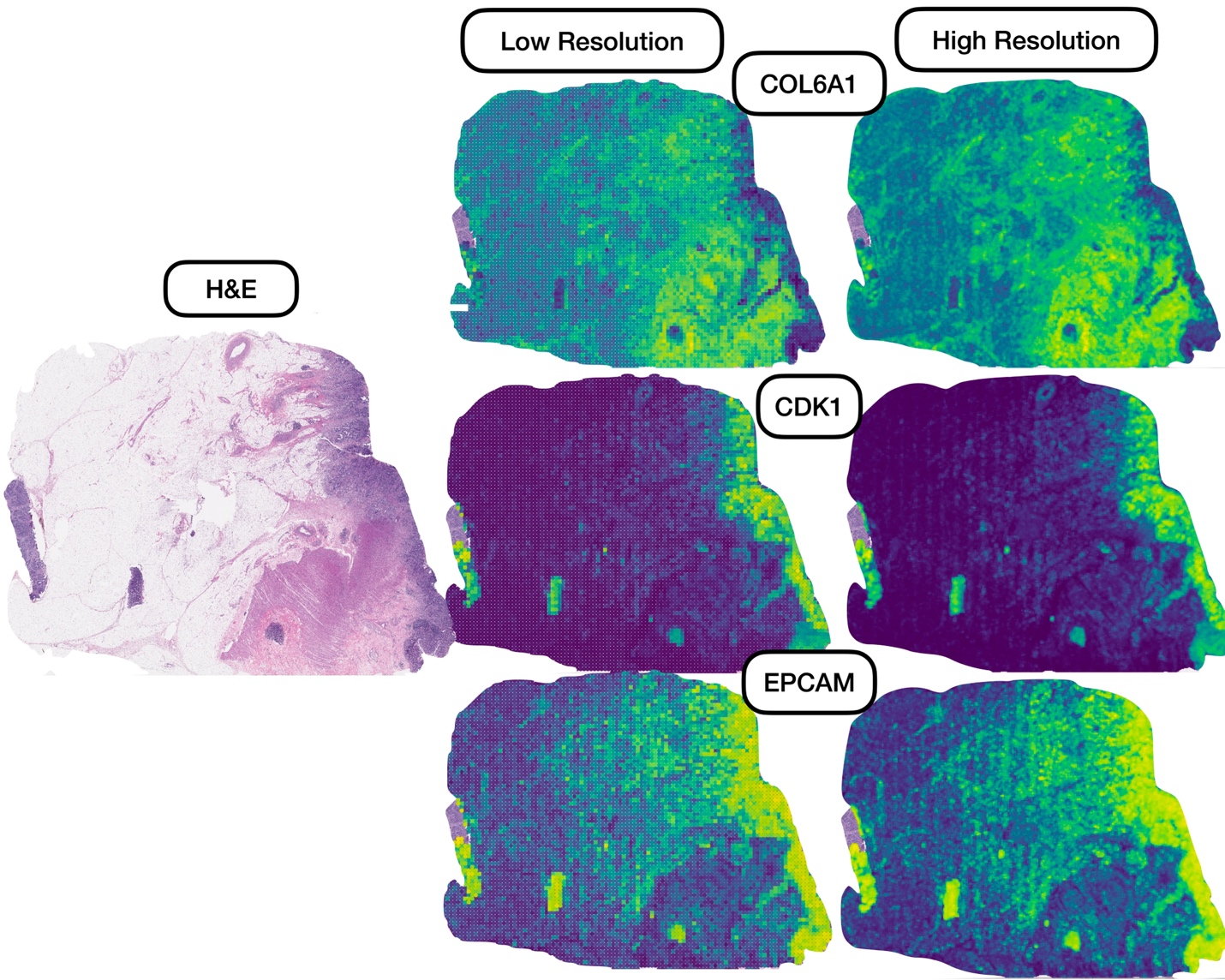
**

**Supplementary Figure 4: High-Resolution Inference of Expression Patterns for a separate T3 CRC patient (held-out slide)–** This figure demonstrates the capacity to extrapolate expression patterns at resolutions surpassing those of the original model training. Utilizing the versatility of imaging features, which can be extracted from any location on the slide, we have conducted inferences at varying resolutions. For low resolution, analysis was performed with non-overlapping 512-pixel strides and patches, whereas high resolution involved overlapping 128-pixel strides, with the same subimage dimensionality. The figure specifically visualizes the expression of COL6A1, indicative of collagen activation, CDK1, a cyclin-dependent kinase associated with tumor activity, and EPCAM, an epithelial cell marker.


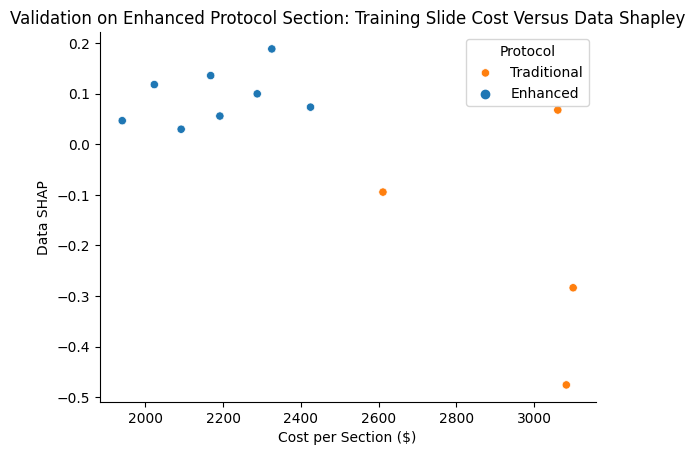


**Supplementary Figure 5: Data Shapley Values for Training Data for Evaluation of Enhanced Protocol on the Validation Slide:** This graph shows the Data Shapley values in relation to the cost per section for training slides processed under both protocols, evaluated against a validation slide prepared with the enhanced protocol. The change in performance, as indicated by Data Shap, demonstrates the enhanced protocol's alignment with clinical workflows, revealing the greater contribution of the training slide to the predictive performance when training and validation protocols match.


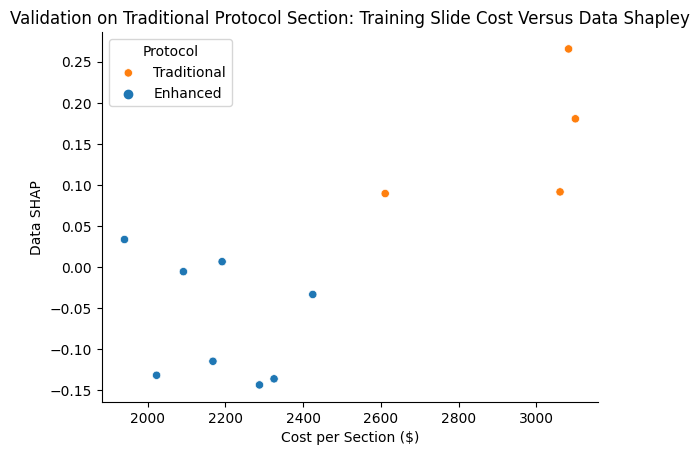


**Supplementary Figure 6: Data Shapley Values for Training Data for Evaluation of Traditional Protocol on the Validation Slide:** Illustrated here are the Data Shapley values versus the cost per section, where the training slides were processed using both protocols but assessed against a traditionally processed validation slide. Notably, the Data Shaleyp values vary, reflecting the differential impact on model performance when the training slides' processing protocol is congruent or incongruent with the validation slide's protocol.

**Supplementary Table 6: Protocol Cost and Data Shapley Values by Training Slide:** Tabulates the number of detectable spots, costs, and Data Shapley values for slides processed using traditional and enhanced protocols. It quantifies the change in algorithmic performance through Data Shapley values when validated against slides prepared with both protocols, highlighting the cost-performance relationship in different processing environments. Sections 5 and 6 were used for validation. Note that the Data Shapley values reported for the enhanced protocol validation slide represent algorithmic benefits for training models on slides that will be evaluated and used in a clinical-grade workflow and thus are of greatest relevance.

| Patient/ Tissue Section | Protocol | Number of Detectable Spots | Slide Cost ($) | Enhanced Validation Slide Data SHAP | Traditional Validation Slide Data SHAP |
| --- | --- | --- | --- | --- | --- |
| 1 | Traditional | 4950 | 3100.29 | -0.28 | 0.18 |
| 2 | Traditional | 4922 | 3082.75 | -0.48 | 0.27 |
| 3 | Traditional | 4887 | 3060.83 | 0.07 | 0.09 |
| 4 | Traditional | 4169 | 2611.13 | -0.09 | 0.09 |
| 7 | Enhanced | 7696 | 2424.83 | 0.07 | -0.03 |
| 8 | Enhanced | 6640 | 2092.11 | 0.03 | -0.01 |
| 9 | Enhanced | 6956 | 2191.67 | 0.06 | 0.01 |
| 10 | Enhanced | 7380 | 2325.26 | 0.19 | -0.14 |
| 11 | Enhanced | 6881 | 2168.04 | 0.14 | -0.11 |
| 12 | Enhanced | 6421 | 2023.10 | 0.12 | -0.13 |
| 13 | Enhanced | 7261 | 2287.77 | 0.10 | -0.14 |
| 14 | Enhanced | 6159 | 1940.55 | 0.05 | 0.03 |

**Supplementary Table 7: Comparison between protocols for Data Shapley values and cost**: Results from Mann-Whitney U-tests comparing training slides processed using the traditional and enhanced protocols across various metrics: specimen assaying costs, Data Shapley values for validation slides processed with each protocol, and Data Shapley-to-cost ratios. Rank biserial correlations and Mann-Whitney p-values provide insight into the effect sizes and statistical significance of the observed differences between protocols.

| Comparison | RBC (Traditional to Enhanced) | P-value |
| --- | --- | --- |
| Cost | -1 | 0.004 |
| Enhanced Validation Slide: Data Shapley | 0.8125 | 0.028 |
| Traditional Validation Slide: Data Shapley | -1 | 0.004 |
| Enhanced Validation Slide: Data Shapley-to-Cost Ratio | 0.9375 | 0.008 |
| Traditional Validation Slide: Data Shapley-to-Cost Ratio | -1 | 0.004 |

**Supplementary Data 1: Reactome pathways corresponding to top 25 genes predicted with greatest performance (spearman) for combination of traditional/enhanced protocol slides.** See suppl_data_1.xlsx.

**References**

1. Fatemi M, Feng E, Sharma C, et al. Inferring spatial transcriptomics markers from whole slide images to characterize metastasis-related spatial heterogeneity of colorectal tumors: A pilot study. Journal of Pathology Informatics 2023; 100308

2. Zeng Y, Wei Z, Yu W, et al. Spatial transcriptomics prediction from histology jointly through Transformer and graph neural networks. Briefings in Bioinformatics 2022; 23:bbac297

3. Pang M, Su K, Li M. Leveraging information in spatial transcriptomics to predict super-resolution gene expression from histology images in tumors. 2021; 2021.11.28.470212

4. He B, Bergenstråhle L, Stenbeck L, et al. Integrating spatial gene expression and breast tumour morphology via deep learning. Nat Biomed Eng 2020; 4:827–834

5. Xie R, Pang K, Chung S, et al. Spatially Resolved Gene Expression Prediction from Histology Images via Bi-modal Contrastive Learning. Advances in Neural Information Processing Systems 2023; 36:70626–70637

6. Jiang Y, Xie J, Tan X, et al. Generalization of deep learning models for predicting spatial gene expression profiles using histology images: A breast cancer case study. 2023; 2023.09.20.558624

7. Srinivasan G, Davis MJ, LeBoeuf MR, et al. Potential to Enhance Large Scale Molecular Assessments of Skin Photoaging through Virtual Inference of Spatial Transcriptomics from Routine Staining. Biocomputing 2024 2023; 477–491

8. Monjo T, Koido M, Nagasawa S, et al. Efficient prediction of a spatial transcriptomics profile better characterizes breast cancer tissue sections without costly experimentation. Scientific reports 2022; 12:1–12

9. Rahaman MM, Millar EKA, Meijering E. Breast cancer histopathology image-based gene expression prediction using spatial transcriptomics data and deep learning. Sci Rep 2023; 13:13604

10. Jia Y, Liu J, Chen L, et al. THItoGene: a deep learning method for predicting spatial transcriptomics from histological images. Briefings in Bioinformatics 2024; 25:bbad464

11. Fatemi MY, Lu Y, Sharma C, et al. Feasibility of Inferring Spatial Transcriptomics from Single-Cell Histological Patterns for Studying Colon Cancer Tumor Heterogeneity. 2023; 2023.10.09.23296701

12. Levy J, Haudenschild C, Barwick C, et al. Topological Feature Extraction and Visualization of Whole Slide Images using Graph Neural Networks. Pac Symp Biocomput 2021; 285–296

13. Karin J, Bornfeld Y, Nitzan M. scPrisma infers, filters and enhances topological signals in single-cell data using spectral template matching. Nat Biotechnol 2023; 41:1645–1654

14. Huynh T, Cang Z. Topological and geometric analysis of cell states in single-cell transcriptomic data. Briefings in Bioinformatics 2024; 25:bbae176

15. Rizvi AH, Camara PG, Kandror EK, et al. Single-cell topological RNA-Seq analysis reveals insights into cellular differentiation and development. Nat Biotechnol 2017; 35:551–560

16. Wang T, Johnson T, Jie Z, et al. Topological Methods for Visualization and Analysis of High Dimensional Single-Cell RNA Sequencing Data. Pacific Symposium on Biocomputing 2019; 24:350–361
